# Rod-Shaped Mesoporous Zinc-Containing Bioactive Glass Nanoparticles: Structural, Physico-Chemical, Antioxidant, and Immuno-Regulation Properties

**DOI:** 10.3390/antiox13070875

**Published:** 2024-07-21

**Authors:** Xiuan Zhu, Wenjie Wen, Jingjing Yan, Yuran Wang, Rumeng Wang, Xiang Ma, Dandan Ren, Kai Zheng, Chao Deng, Jue Zhang

**Affiliations:** 1 Anhui Province Engineering Research Center for Dental Materials and Application, School of Stomatology, Binjiang Campus, Wannan Medical College, No. 22, West Wenchang Road, Yijiang District, Wuhu 241002, China; zhuxiuan@wnmc.edu.cn (X.Z.); 20230025@wnmc.edu.cn (W.W.); 20229566@stu.wnmc.edu.cn (J.Y.); 21105010120@stu.wnmc.edu.cn (Y.W.); 21105010119@stu.wnmc.edu.cn (R.W.); 20105010063@stu.wnmc.edu.cn (X.M.); 20230035@wnmc.edu.cn (D.R.); 2 Jiangsu Province Engineering Research Center of Stomatological Translational Medicine, Nanjing Medical University, Nanjing 210029, China; kaizheng@njmu.edu.cn

**Keywords:** bioactive glass, rod-shaped nanoparticles, radical-scavenging activity, macrophage polarization

## Abstract

Bioactive glass nanoparticles (BGNs) are applied widely in tissue regeneration. Varied micro/nanostructures and components of BGNs have been designed for different applications. In the present study, nanorod-shaped mesoporous zinc-containing bioactive glass nanoparticles (ZnRBGNs) were designed and developed to form the bioactive content of composite materials for hard/soft tissue repair and regeneration. The nanostructure and components of the ZnRBGNs were characterized, as were their cytocompatibility and radical-scavenging activity in the presence/absence of cells and their ability to modulate macrophage polarization. The ZnRBGNs possessed a uniform rod shape (length ≈ 500 nm; width ≈ 150 nm) with a mesoporous structure (diameter ≈ 2.4 nm). The leaching liquid of the nanorods at a concentration below 0.5 mg/mL resulted in no cytotoxicity. More significant improvements in the antioxidant and M1-polarization-inhibiting effects and the promotion of M2 polarization were found when culturing the cells with the ZnRBGNs compared to when culturing them with the RBGNs. The doping of the Zn element in RBGNs may lead to improved antioxidant and anti-inflammatory effects, which may be beneficial in tissue regeneration/repair.

## 1. Introduction

Bioactive glass (BG) has been employed in clinics for more than 50 years since it was first prepared by Larry Hench and co-workers. It belongs to the SiO_2_-Na_2_O-CaO-P_2_O_5_ system [1]. Until now, BG with different components and micro/nanostructures has been prepared for different tissue regeneration/repair applications [2]. 

Mesoporous bioactive glass nanoparticles (MBGNs) with a uniform shape (sphere/rod) have attracted more attention due to their mesoporous structure, uniform shape, and high specific surface area compared with those of traditional BG [3]. The characteristics of the MBGNs mentioned above enable the efficient exchange of therapeutic ions and drugs/functional molecules loaded into Si-O-Si or the porous structure. Plenty of studies have used MBGNs as drug carriers, orthopedic implant coating materials, enhanced bioactive hydrogels, etc. [4,5].

All these materials usually show multifunctional biomedical properties, partly due to the ionic dissolution products of BG [6]. More and more researchers are showing that the ionic dissolution products of MBGNs during degradation stimulate biological functions, such as osteogenesis, angiogenesis, and antibacterial and antioxidant processes, as well as impacting the immune system by inducing the release of specific growth factors or gene expression in different types of cells [7,8,9]. 

Some recent studies found that the immune system plays an important role in the tissue regeneration process [10,11]. Macrophages play a central role in inflammation. Different phenotypes may be displayed in response to different environmental stimuli [12,13]. The polarization of macrophages (M1 and M2 phenotypes) is very important throughout the regeneration process [14,15]. 

The release of reactive oxygen species (ROS) from cells also has beneficial effects on tissue repair [16]. A long-term and relatively large increase in the quantity of ROS induces damage to vital cellular macromolecules such as DNA, proteins, and lipids. Additionally, a range of reactive species can stimulate an intracellular signaling cascade that increases pro-inflammatory gene expression [17]. 

The ionic products from the dissolution of bioactive glass showed a great influence on macrophage polarization and strong antioxidant effects [18,19]. A previous study showed that Mg and Si ions dissolved from Mg-containing bioactive glass nanoparticles can regulate the polarization (from M1 to M2) of macrophages and promote osteogenesis [20]. Zinc (Zn) is an important element in the physiological environment. It is the key mediator of immune reactions, bone matrix mineralization, and bone regeneration [21]. A recent study showed that the incorporation of zinc into silicate-based bioglass evidently promoted osteogenesis in vitro while profoundly suppressing inflammatory reactions and osteoclastogenesis [22]. Yu’s study found that Zn-coated scaffolds resulted in high M1 polarization at 6 h, triggering an M2 response after 24 h [23]. Another study also revealed that Zn can negatively regulate NF-κB signaling by inactivating PPAR-α, A20, IκB kinase-β (IKKβ), and phosphodiesterase (PDE); realize the conversion of M1 macrophages into M2 macrophages; and induce the secretion of a variety of cytokines, thereby regulating tissue regeneration [24]. Zinc is also widely accepted to be an effective antioxidant element [25]. Cerium-doped bioactive glass was regarded as a representative of the antioxidant series of bioactive glass, but few studies have focused on the antioxidant properties of Zn-doped bioactive glass [26].

The shape of particles is one of the factors influencing the mechanical properties, the ionic products/drug-dissolving process of the MBGN-based implant, the composite hydrogel, etc. [27]. Many studies have focused on the preparation and properties of spherical mesoporous bioactive glass nanoparticles. Recently, plenty of research has found that nanorods often seem to be more efficient in drug delivery to cancer cells compared to nanospheres [28,29]. Moreover, rod-shaped nanoparticle-reinforced nanocomposites showed better mechanical properties than those of spherical nanoparticles [30]. However, there has been less research on rod-shaped bioactive glass nanoparticles than on spherical ones. The fabrication methods, the immune-modulating functions, and the radical-scavenging activity of the rod-shaped Zn-containing bioactive glass nanoparticles still need to be explored.

In this study, rod-shaped zinc-containing mesoporous bioactive glass nanoparticles (ZnRBGNs) were synthesized via a sol–gel method. Zinc ions were doped onto a glass structure using a sintering method. BGNs with different contents of zinc ions were fabricated. The structure and composite of the particles were studied. The cytocompatibility of the particles was evaluated by co-culturing them with L929 cells. Furthermore, the macrophage polarization and radical-scavenging activity induced by the ZnRBGNs in the presence/absence of cells in vitro were explored.

## 2. Materials and Methods

### 2.1. Preparation of Zn-Containing Rod-Shaped Mesoporous Bioactive Glass Nanoparticles (ZnRBGNs)

The ZnRBGNs were fabricated using a sol–gel method according to previous studies [25]. Briefly, 0.15 g of cetyltrimethylammonium bromide (CTAB) was dissolved into 18 mL of ddH_2_O at 60 °C. After the solution had been stirred for 20 min at 400 r/min, 1.5 mL of aqueous ammonia (NH_3_-H_2_O) was dropped into the solution and left to react for 10 min. A total of 0.31 mL of tetraethyl orthosilicate (TEOS) was added to the abovementioned solution. After the reaction had proceeded for 4 h, the products were collected through centrifugation at 8000 rpm for 10 min and then washed with water twice and ethanol once. After drying in an air blast drying oven at 60 °C overnight, the products were dispersed into a zinc acetate solution (1M) and stirred at 300 rpm for 0 h, 2 h, 8 h, and 24 h. Then, the powder was collected through centrifugation at 8000 rpm for 10 min and then washed with water twice and ethanol once. After sintering in a muffle furnace at 700 °C for 4 h, samples named 0h-ZnRBGN, 2h-ZnRBGN, 8h-ZnRBGN, and 24h-ZnRBGN were obtained. All the chemicals were purchased from Shanghai Macklin Biochemical Technology Co., LTD. (Shanghai, China).

### 2.2. Characterization of the Physical and Chemical Properties of the Samples

The surface morphologies of 0h-ZnRBGN, 2h-ZnRBGN, 8h-ZnRBGN, and 24h-ZnRBGN were tested using a field emission scanning electron microscope (FE-SEM, Regulus 8100, Hitachi, Tokyo, Japan) at an accelerating voltage of 5 kV. Prior to SEM characterization, the nanoparticles were sputter-coated with gold for 30 s using a Quorum SC7640 high-resolution sputter coater to maintain an electrically conductive path. The nanostructure and composition of the different samples were observed using a transmission electron microscope (TEM, X-Max, Oxford Instrument, Abingdon, UK) equipped with an energy-dispersive spectrometer (EDS) at an accelerating voltage of 100 kV. The surface area and pore size of the samples were characterized using a volumetric adsorption analyzer (ASAP 2460, Micromeritics) based on the Brunauer–Emmett–Teller (BET) method. The specific surface area, pore size, and total pore volume were calculated using the Barrett–Joyner–Halenda (BJH) method using the desorption isotherm branch. The chemical structures of all the ZnRBGNs were characterized using a Fourier-transform infrared spectrometer (ATR-FTIR, TENSOR 27, Bruker, Billerica, MA, USA) at wavenumbers ranging from 400 to 2000 cm^−1^ with a resolution of 4 cm^−1^. X-ray photoelectron spectroscopy (XPS, ESCALAB 250xi, Thermo Fisher Scientific, Waltham, MA, USA) was conducted to measure the composition and states of the Si, O, and Zn elements. The structure of the glass phase was determined using X-ray diffraction (XRD, Rigaku, Smartlab) with Cu Kα radiation (40 mA; 40 kV; 2θ range: 10−80°; scanning speed: 2°/min).

### 2.3. Cytocompatibility Evaluation In Vitro

#### 2.3.1. Cell Culture

The cytocompatibility of the samples was evaluated by co-culturing them with L929 (American Type Culture Collection; Manassas, VA, USA). The effects on macrophage polarization were evaluated by co-culturing the samples with RAW 264.7 (American Type Culture Collection; Manassas, VA, USA). The cells were cultured in Dulbecco’s Minimum Essential Medium (DMEM; Tianjin Songyang, China) supplemented with 10% fetal bovine serum (FBS) and 1% penicillin–streptomycin (Sigma-Aldrich, St. Louis, MI, USA) at 37 °C in a humidified 5% CO_2_ incubator. The medium was changed every 2 days. 

#### 2.3.2. Preparation of Leaching Solution of Samples

The leaching solution of the samples was prepared to study the cytocompatibility and polarization of the macrophages. The leaching solutions were prepared by dispersing the 0h-ZnRBGN, 2h-ZnRBGN, 8h-ZnRBGN, and 24h-ZnRBGN samples in DMEM at 0.01 mg/mL, 0.1 mg/mL, 0.5 mg/mL, and 1 mg/mL at 37 °C with 200 rpm shaking for 24 h. After filtering with a 0.22-micron filter head, 10% FBS and 1% penicillin–streptomycin were added to the above solution for the following co-culture.

#### 2.3.3. Cytotoxicity and Proliferation

The cytotoxicity and proliferation were evaluated according to a previous study [16]. Briefly, L929 cells were cultured on 24-well plates at 2 × 10^4^ per well overnight for the adhesion of cells. Then, the culture medium was changed to a leaching solution, and after 24 h of co-culturing, the medium was aspirated, and 300 μL of 10% CCK-8 solution was added. The plate was incubated at 37 °C for 2 h, and the OD value of the supernatant was determined using a microplate reader (Biotek, Cytation3, Rocklin, CA, USA) at 450 nm. 

#### 2.3.4. EdU Assay

The EdU cell proliferation kit (E-Click EdU Cell Proliferation Imaging Assay Kit, Elabscience Biotechnology Co., Ltd. Wuhan, China) was used to test cell proliferation. The experiment was conducted according to a previous study [31]. Briefly, the L929 cells seeded in 24-well plates (2 × 10^4^ cells/well) were cultured for 24 h. Then, the cells were treated using an Edu solution; fixed with 4% paraformaldehyde, followed by a treatment with TritonX-100; and then stained using Dapi solution. The EdU-positive cell rate was analyzed using fluorescence microscopy (Olympus, IX73, Shinjuku, Japan) and Image J (version: 2.14.0).

#### 2.3.5. Live–Dead Assay

The viability of L929 co-cultured with the leaching medium (0.1 mg/mL) was qualitatively evaluated using live–dead staining. The living cells were stained with calcein acetoxymethyl ester (calcein AM), while the dead cells were stained with propidium iodide (Shanghai ShengGong, Shanghai, China) according to a previous study [32]. Briefly, the cells were collected through centrifugation at 800 rpm after co-culturing them with the leaching medium for 24 h. Then, the samples were incubated with a staining solution containing 0.4% calcein AM and 0.5% propidium iodide in HBSS at 37 °C in a humidified 5% CO_2_ incubator for 30 min. The stained samples were observed using a fluorescence microscope (Olympus, IX73, Shinjuku, Japan), and the data were analyzed using Image J (version: 2.14.0).

#### 2.3.6. Flow Cytometry

Flow cytometry was used to identify the M1 (CD86) and M2 (CD206) macrophage surface markers according to a previous study [16]. The RAW 264.7 cells were plated (1 × 10^5^ cells/well) and cultured with the leaching medium for 3 days. Then, the cells were gently scraped and centrifuged at 300× *g* for 5 min, followed by two rinses with PBS and blocking with 1% bovine serum albumin (BSA) for 30 min. Following this treatment, the cells were labeled with Brilliant Violet 421 (BV421)-conjugated CD206 and Alexa Fluor 647 (AF647)-conjugated CD86 (BD Pharminge, San Diego, CA, USA). A flow cytometer (BD, FACSVerse, San Diego, CA, USA) was used to analyze 500 μL suspensions of the cells.

### 2.4. Antioxidant Effect Evaluation

#### 2.4.1. ROS (Reactive Oxygen Species) Generation Assay 

The quantity of ROS in L929 was measured using the fluorogenic probe 2′,7′-dichlorofluorescein diacetate (DCFH-DA) in the ROS generation assay kit (UElandy Inc., Suzhou, China) [33]. In brief, 1 × 10^4^ cells were seeded into a 96-well plate and incubated for 24 h. Subsequently, the medium was changed to a leaching medium. After incubation for 24 h, the cells were washed and exposed to DCFH-DA (30 μM) for 90 min. After being washed with PBS 3 times, the cells were re-suspended in DMEM without FBS. The cells cultured with a normal medium were the negative control. The cells were observed using a fluorescence microscope (Olympus, IX73, Japan), and the fluorescence intensity was analyzed using Image J (version: 2.14.0).

#### 2.4.2. DPPH Assay

The radical-scavenging capacities of 0h-ZnRBGN and 8h-ZnRBGN were evaluated using the DPPH (1,1-Diphenyl-2-picrylhydrazyl radical 2,2-Diphenyl-1-(2,4,6-trinitrophenyl) hydrazyl) radical-scavenging method [34]. Concentrations of 5 mg/mL of 0h-ZnRBGN and 8h-ZnRBGN were dispersed into a DMSO solution of DPPH (200 µM, Sigma Aldrich) and then incubated for 3 h; after this, the absorbance was recorded at 517 nm using a microplate reader (Biotek, Cytation3, USA).

All the results were calculated using the following formula.

Radical-scavenging activity (%) = [1 − (Ae − Ag)/Ac] × 100

Ac: Absorbance of DPPH solution in EtOH at 517 nm

Ag: Absorbance of the samples solutions in EtOH at 517 nm

Ae: Absorbance of DPPH and samples solution in EtOH at 517 nm

### 2.5. Statistical Analysis

All the experiments were performed using biological replicates, and the data are expressed as the mean ± standard deviation. One-way/two-way analysis of variance (ANOVA) was used to analyze the significance of the differences between the samples. Statistical analyses were performed using GraphPad Prism 9 software (GraphPad, Inc., La Jolla, CA, USA). *p* values < 0.05 were considered to indicate statistically significant results.

## 3. Results

### 3.1. Characterization of the Physical and Chemical Properties of the Samples

#### 3.1.1. The Morphology and Composition of ZnRBGNs

**Figure 1:**
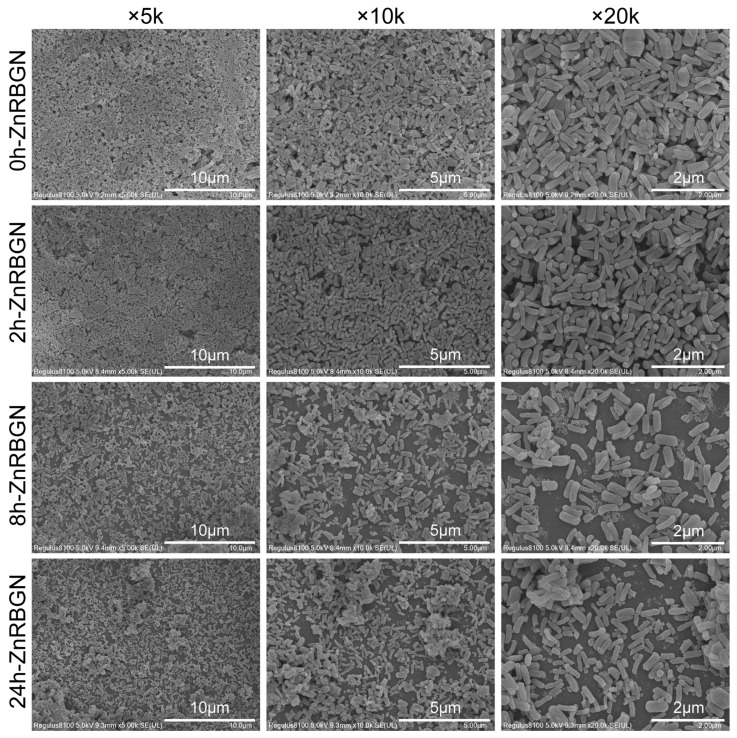
Scanning electron microscopy (SEM) images of 0h-ZnRBGN, 2h-ZnRBGN, 8h-ZnRBGN, and 24h-ZnRBGN (×5 k, ×10 k, and ×20 k).

**Figure 2:**
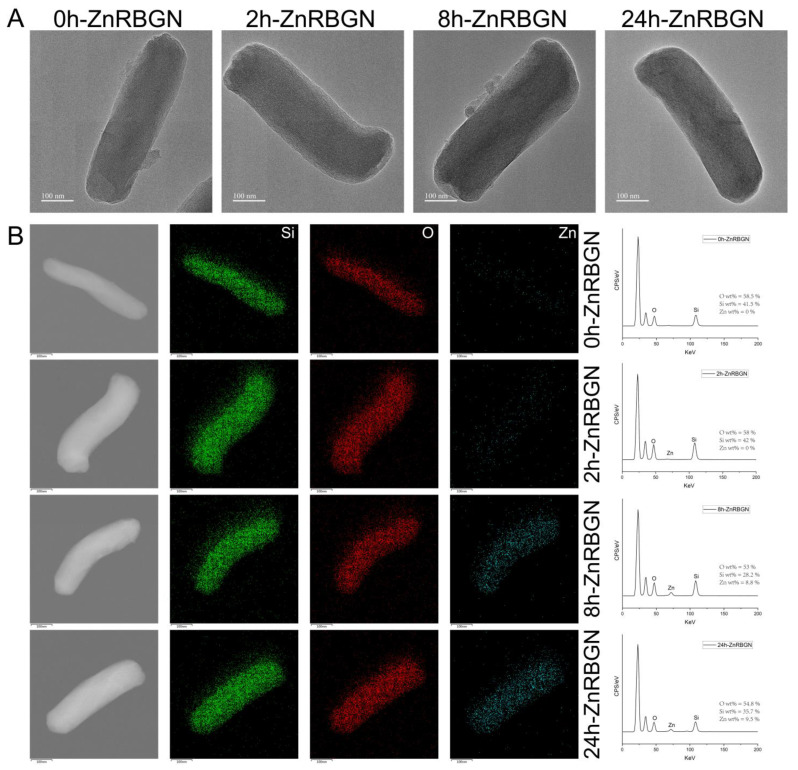
(**A**) Transmission electron microscope (TEM) images of 0h-ZnRBGN, 2h-ZnBGN, 8h-ZnRBGN, and 24h-ZnRBGN. (**B**) Energy spectrum analysis and element distribution (Green pots represented distribution of Si element, red for O and blue for Zn) of 0h-ZnRBGN, 2h-ZnRBGN, 8h-ZnRBGN, and 24h-ZnRBGN.

In this study, rod-shaped zinc-containing mesoporous bioactive glass nanoparticles (ZnRBGNs) were prepared using the sol–gel method in an alkaline environment. Zn ions were incorporated into the structure using the immersing–sintering method. The micro/nanostructure of ZnRBGN was observed using TEM and SEM. According to the SEM results (Figure 1), the synthesized rod-shaped bioglass nanoparticles (ZnRBGNs) were monodispersed and showed a rod-shaped morphology, which is a typical feature of silica-based particles synthesized using the Stöber method under alkaline conditions. After doping the zinc element through immersion in a Zn solution for different durations (2 h, 8 h, and 24 h), the samples were still rod-shaped. The nanorods were about 500 nm in length and 150 nm in diameter, which is in accordance with the transmission electron microscopy results (TEM, Figure 2A). All the samples showed a similar rod-shaped morphology, indicating that the doping of Zn did not affect the structure of the bioglass nanoparticles.

Similar to the SEM results, the TEM images showed a rod-shaped morphology with a porous structure for all the ZnRBGNs, which is the typical morphology when using the template method of CTAB. The doping of the Zn element did not significantly change the porous structure. The energy spectrum was analyzed using TEM-EDS at the same time. The elemental distributions of Si and Zn were studied; the green parts reflect the distribution of Si, the red parts indicate O, and the blue parts denote the distribution of the Zn element. The results showed that the Zn composition was improved with the immersion time. The Zn composition of 2h-ZnRBGN could not be measured using TEM-EDS, which showed that the minimum reaction time should be more than 2 h. The Zn composition of 8h-ZnRBGN (Zn wt % = 8.8%) was similar to that of 24h-ZnRBGN (Zn wt % = 9.5%), which indicated that maximum reaction time was about 8 h for Zn doping. Based on the above results, the suitable immersion time in the Zn solution was 8 h. Additionally, the monodispersed nanorods were prepared, and the doping of zinc did not change the rods’ morphology.

**Figure 3:**
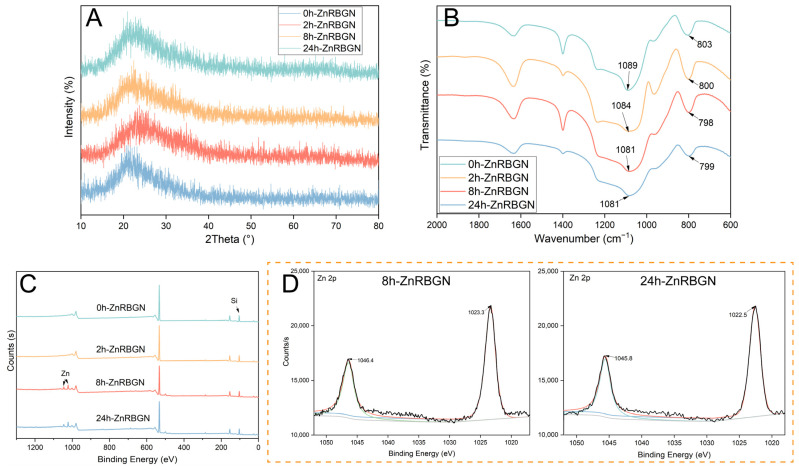
(**A**) XRD pattern, (**B**) FTIR, (**C**) XPS wide scan, and (**D**) narrow scan analyses of 0h-ZnRBGN, 2h-ZnBGN, 8h-ZnRBGN, and 24h-ZnRBGN.

To study the structure and composition of the samples, XRD, FTIR, and XPS analyses were conducted. A broad peak from about 2theta = 15° to 25° can be seen in the XRD patterns (Figure 3A) of all the samples, which is a typical characteristic of amorphous silicate materials. No significant differences could be found in the XRD patterns of all the samples. The results indicated that the amorphous structure of glass was maintained with the doping of Zn ions, and no Zn crystalline phase developed. The FTIR spectra of the different ZnRBGNs showed the characteristic bands of silicate glass (Figure 3B). The wide band located in the range of 1000–1200 cm^−1^ is attributed to Si-O-Si symmetric stretching vibration. The bands at 806 and 474 cm^−1^ are ascribed to the symmetric stretching and rocking of Si-O, respectively. All the above results indicate that the blending of Zn ions did not affect the amorphous silica glass structure. 

XPS data for all the samples were obtained for the evaluation of the oxidation state of metals, the elemental composition, and the distribution of species (Figure 3C,D). The results showed that the elements distributed in the sample 0h-ZnRBGN were Si and O (Figure 3C), while the elements in 8h-ZnRBGN and 24h-ZnRBGN were Zn, Si, and O. Similar to the results of EDS, peaks corresponding to Zn in 2h-ZnRBGN were too weak to find. Two peaks appeared for 8h-ZnRBGN and 24h-RBGN (Figure 3D) at 1023 and 1046 eV as a result of the 2p orbital splitting of Zn^2+^ of ZnO. Moreover, the Zn 2p peaks are slightly higher, by 1 eV, than the peak of pure ZnO without a host, indicating that there was some Zn interaction with the Si atoms of the host [35]. The composition of Zn in the nanorods improved with immersion during the 8 h. The amorphous silicate structure was maintained after the doping of Zn.

#### 3.1.2. The Mesoporous Structural Analysis of ZnRBGN

**Figure 4:**
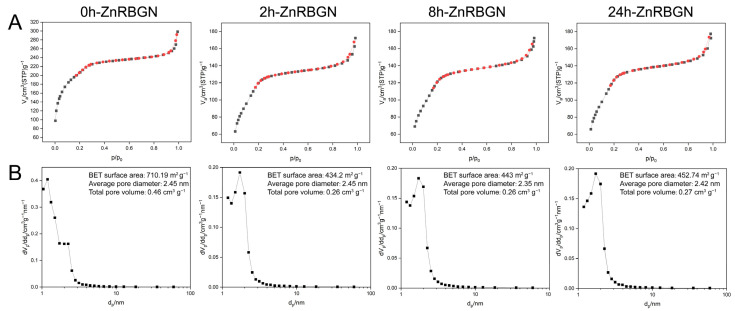
N_2_ adsorption–desorption isotherms (**A**) and pore size distributions (**B**) of 0h-ZnRBGN, 2h-ZnRBGN, 8h-ZnRBGN, and 24h-ZnRBGN.

A classical type IV isotherm with an H1-type hysteresis can be seen in the N_2_ isotherms of all the samples, corresponding to typical mesoporous materials according to the International Union of Pure and Applied Chemistry (IUPAC) classification (Figure 4A). The specific surface areas of 0h-ZnRBGN, 2h-ZnRBGN, 8h-ZnRBGN, and 24h-ZnRBGN were 710.19 m^2^g^−1^, 434.2 m^2^g^−1^, 443 m^2^g^−1^, and 452.74 m^2^g^−1^; the total pore volumes of 0h-ZnRBGN, 2h-ZnRBGN, 8h-ZnRBGN, and 24h-ZnRBGN were 0.46 cm^3^g^−1^, 0.26 cm^3^g^−1^, 0.26 cm^3^g^−1^, and 0.27 cm^3^g^−1^ (Figure 4B). The decreases in the total pore volume and specific surface area may have been due to the immersion treatment. However, the treatment did not affect the average pore diameter (about 2.4 nm). 

### 3.2. Cytocompatibility Evaluation In Vitro

**Figure 5:**
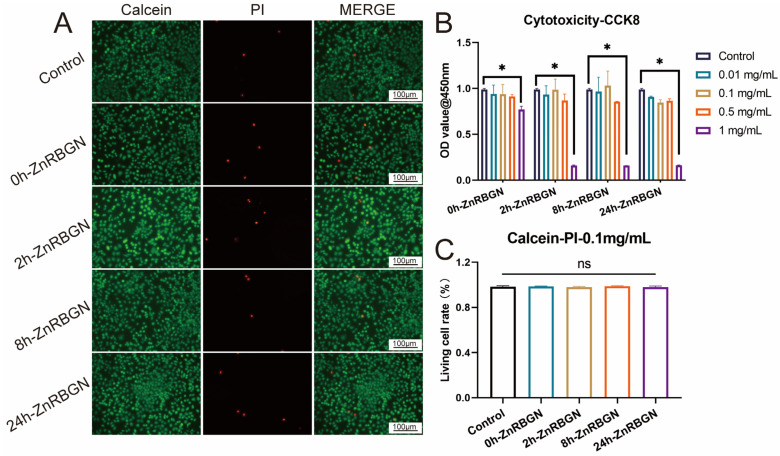
The cytotoxicity evaluation for 0h-ZnRBGN, 2h-ZnRBGN, 8h-ZnRBGN, and 24h-ZnRBGN. (**A**) Live and dead staining. Fluorescence imaging of calcein and PI staining of samples in 0.1 mg/mL. (**B**) OD value of L929 culture with extract of different samples in 0.01, 0.1, 0.5, and 1 mg/mL. (**C**) Quantitative evaluation of live and dead staining. * *p* < 0.05, “ns” means no significant difference.

The L929 cells were used to evaluate the cytotoxicity of 0h-ZnRBGN, 2h-ZnRBGN, 8h-ZnRBGN, and 24h-ZnRBGN and their effects on proliferation. Concentrations of 0.01 mg/mL, 0.1 mg/mL, 0.5 mg/mL, and 1 mg/mL leaching solution were co-cultured with L929 for 24 h to evaluate the cytotoxicity of the samples. The results were as follows: (a) the extracts showed no significant cytotoxicity at 0.5 mg/mL; (b) all the 1 mg/mL extracts showed significantly more cytotoxicity than the control. The following co-culturing experiment was conducted with 0.1 mg/mL extracts of all the samples (Figure 5B). 

Figure 5A shows live/dead staining images of L929s after 24 h of co-culturing with 0.1 mg/mL extracts of the different samples. Compared with the control, all the ZnRBGNs showed no significant toxicity (Figure 5A). The proportion of dead cells (marked in red) was about 2% in all the samples (Figure 5C). 

**Figure 6:**
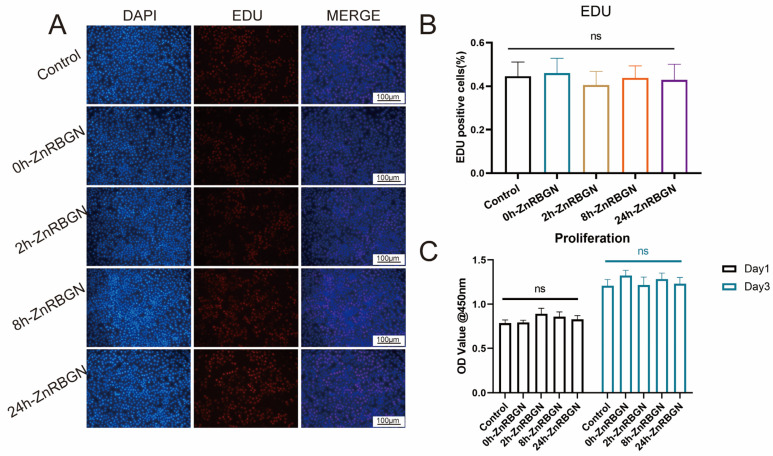
Evaluation of the effects of 0.1 mg/mL extracts of 0h-ZnRBGN, 2h-ZnRBGN, 8h-ZnRBGN, and 24h-ZnRBGN proliferation. (**A**) EDU and Dapi staining. The EDU fluorescence imaging and Dapi staining for the 0.1 mg/mL samples. (**B**) The quantitative evaluation of staining. (**C**) The OD values of the L929 culture with 0.1 mg/mL extracts of the different samples on days 1 and 3, “ns” means no significant difference.

The effect of 0h-ZnRBGN, 2h-ZnRBGN, 8h-ZnRBGN, and 24h-ZnRBGN on the proliferation of L929 cells was determined using CCK-8 and EDU assays (Figure 6). There was no significant difference in cell proliferation between the experimental and control groups on days 1 and 3. The results revealed that none of the 0.1 mg/mL extracts of the samples could promote the proliferation of L929 cells. 

**Figure 7:**
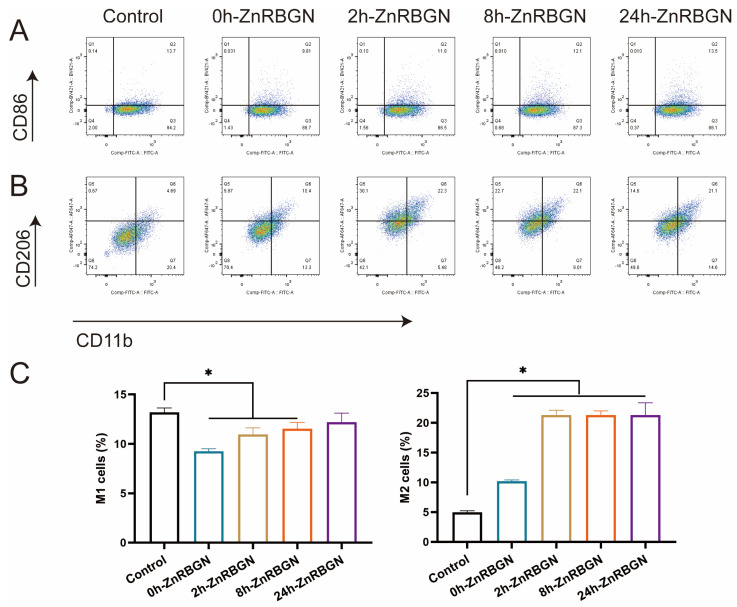
The flow cytometry analysis of macrophage surface marker expression; the fluorescence intensity of CD86 (**A**) and CD206 (**B**) of the RAW 264.7 cells cultured with 0h-ZnRBGN, 2h-ZnRBGN, 8h-ZnRBGN, and 24h-ZnRBGN. (**C**) The macrophage populations of M1 (CD86)- and M2-polarized (CD206) RAW 264.7 cells with 0h-ZnRBGN, 2h-ZnRBGN, 8h-ZnRBGN, and 24h-ZnRBGN. * *p* < 0.05.

To investigate the polarization of macrophages in response to the extracts of 0h-ZnRBGN, 2h-ZnRBGN, 8h-ZnRBGN, and 24h-ZnRBGN, the proportion of M1- and M2-polarized RAW 264.7 cells was examined using flow cytometry (Figure 7). 

The fluorescence intensities of the M1 marker CD86 and the M2 marker CD206 were calculated (Figure 7C). The results revealed that 0h-ZnRBGN and 2h-ZnRBGN suppressed M1 polarization; this may have been due to the exit of the Si element, even though the Zn element promotes M1 polarization. Culturing with 2h-ZnRBGN, 8h-ZnRBGN, and 24h-ZnRBGN significantly promoted M2 polarization, while that with 0h-ZnRBGN did not result in a significant difference from the control. This may illustrate that the exit of Zn may facilitate the M2 polarization of RAW 264.7, while the exit of Si may have no effect on such polarization. Taken together, these results suggested that 2h-ZnRBGN had a more pronounced anti-inflammatory effect than that of the other group MBG, and 2h-ZnRBGN, 8h-ZnRBGN, and 24h-ZnRBGN promoted the conversion of RAW 264.7 to the M2 phenotype.

### 3.3. Antioxidant Effect Evaluation 

**Figure 8:**
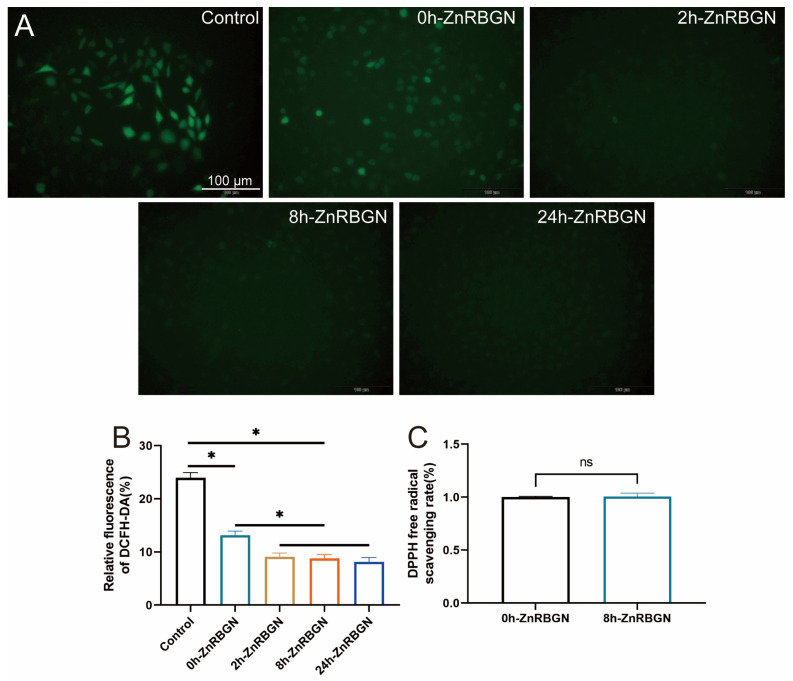
Antioxidant effect analysis. (**A**) ROS generation assay analysis. Images of the cells cultured with the leaching medium (green fluorescence represents ROS) and quantitative analysis (**B**). (**C**) The DPPH free radical-scavenging rates with 0h-ZnRBGN and 8h-ZnRBGN. * *p* < 0.05, “ns” means no significant difference.

In this work, L929 cells were cultured to evaluate the capacity of rod-shaped nanoparticles to scavenge ROS. The control group showed the highest ROS level, the RBGNs without the Zn element showed a lower ROS level, and all the Zn-containing RBGNs showed the lowest ROS level. No significant differences among the groups of 2h-ZnRBGN, 8h-ZnRBGN, and 24h-ZnRBGN could be found (Figure 8A,B). A DPPH assay is generally used to measure non-enzymatic antioxidant activity. The ZnRBGNs do not have a direct scavenging capacity at 5 mg/mL (Figure 8C).

## 4. Discussion

Usually, bioglass nanoparticles are formed into a composite with a polymer substrate to improve hydrophilicity, biocompatibility, osteogenesis, and immune modulation. Bioglass can establish a characteristic environment through the exchange of different types of ions. Different kinds of elements, such as Cu, Mg, Zn, and Ce, were incorporated into the amorphous structure [36]. These ions could be incorporated into the glass structure through the one-step [37] or two-step [38] molding of glass nanoparticles. In our research, the two-step molding process was used for the preparation of the rod-shaped zinc-doped bioactive glass nanoparticles due to the strong alkaline environment, which may have led to the changes in rod shape and mesoporous structure.

Most of the bioactive glass nanoparticles studied were prepared using the Stöber method and had a spherical shape. The rod-shaped mesoporous silica nanoparticles were more effective at delivering drugs to cancer cells than the spherical nanoparticles were. Moreover, the rod-shaped mesoporous silica nanoparticles had better mechanical properties when used as composite materials compared with those of the spherical particles [39]. Considering the application of bioactive glass nanoparticles, rod-shaped bioactive glass nanoparticles also need to be more intensively researched.

This study successfully synthesized rod-shaped mesoporous zinc-containing bioactive glass nanoparticles (ZnRBGNs) and characterized their structure, cytocompatibility, antioxidant properties, and immune modulation capabilities. These findings highlight the significant potential of ZnRBGNs in biomedical applications, particularly in tissue regeneration. The ZnRBGNs retained a rod-shaped morphology with a uniform size distribution and mesoporous structure, which is essential for enhancing ion exchange and drug-delivery capabilities. However, interestingly, we observed a significant decrease in the BET surface area and total pore volume while the pore diameter was maintained. This may have been due to immersion in the zinc acetate solution; some pore structures of the nanoparticles could have been corroded by the environment.

However, the dispersibility of the nanorods must still be improved. We observed the aggregation of nanorods after dispersing them in the liquid environment. The search for new methods to fabricate new nanocomposites is ongoing.

The successful incorporation of zinc was confirmed by EDS and XPS analyses, indicating its homogeneous distribution within the silica matrix. Zinc can promote angiogenesis, cell proliferation, and migration, as well as the wound-healing process [40]. However, the application of Zn was limited by its toxicity [41]. The doping of Zn into the structure of bioactive glass could lead to the sustained and controlled release of Zn ions. This incorporation is crucial, as zinc ions play vital roles in various biological processes, including enzyme functions, protein synthesis, and cellular signaling. 

The cytocompatibility assays demonstrated that the ZnRBGNs did not exhibit significant cytotoxicity at the tested concentrations, ensuring their safety for biomedical applications. The extracts of ZnRBGNs had no significant influence on the proliferation of L929 cells. The ZnRBGNs exhibited strong antioxidant properties, as evidenced by the significant reduction in intracellular ROS levels. The antioxidant capacity of ZnRBGNs is particularly beneficial in mitigating oxidative stress, which is a common challenge in tissue repair and regeneration. High-level oxidative stress can damage cellular components and impede the healing process. By scavenging ROS, ZnRBGNs can protect the cells from oxidative damage and create a healthier regenerative environment. The DPPH assay showed that the ZnRBGNs did not obtain antioxidant properties in the absence of cells. The ions of the extracts were not reducible. 

The ability of ZnRBGNs to modulate macrophage polarization is the key finding of this study. The nanoparticles promoted the polarization of macrophages toward the M2 phenotype, which is associated with anti-inflammatory and tissue repair functions, while eliminating the M1 phenotype, which is associated with pro-inflammatory responses. This shift toward M2 polarization is crucial for creating a regenerative microenvironment, as M2 macrophages release anti-inflammatory cytokines and growth factors that support tissue healing and regeneration. The modulation of macrophage behavior by the ZnRBGNs underscores their potential in managing inflammation and enhancing the healing process. The unique combination of properties exhibited by the ZnRBGNs, e.g., toxicity, enhanced cell proliferation, strong antioxidant activity, and favorable immune modulation, makes them highly promising for applications in tissue regeneration. Their ability to simultaneously support cell growth, reduce oxidative stress, and modulate immune responses positions ZnRBGNs as multifunctional agents that can address several critical aspects of the regenerative process. Future research should focus on in vivo studies to further validate the efficacy of ZnRBGNs in tissue regeneration. Investigating the long-term effects and biodegradation profiles of these nanoparticles in living organisms will provide more comprehensive insights into their potential clinical application. Additionally, exploring the incorporation of other therapeutic ions or molecules into ZnRBGNs could enhance their multifunctionality and broaden their application scope. 

## 5. Conclusions

In summary, the rod-shaped mesoporous zinc-containing bioactive glass nanoparticles developed in this study can be used for tissue regeneration applications. The doping of the zinc element did not change the mesoporous and rod-shaped structure of the nanoparticles. A suitable immersion time is about 8 h. ZnRBGNs’ biocompatibility, antioxidant activity, and ability to modulate macrophage polarization highlight their therapeutic promise. These findings pave the way for further research and the development of ZnRBGNs as versatile and effective materials in regenerative medicine.

## Data Availability

All the data sets collected and analyzed during this study are available from the corresponding author upon reasonable request.
